# Side effects of toxic bait formulations on *Diachasmimorpha longicaudata* (Hymenoptera: Braconidae)

**DOI:** 10.1038/s41598-019-49106-z

**Published:** 2019-08-29

**Authors:** Daniel Bernardi, Aline Nondillo, Cléber Antonio Baronio, Lígia Caroline Bortoli, Ruben Machota Junior, Rute Caroline Becker Treptow, Fernanda Carla Santos Geisler, Camila Gauger Neitzke, Dori Edson Nava, Marcos Botton

**Affiliations:** 1 Universidade Federal de Pelotas, Departamento de Fitossanidade, Caixa Postal 354, CEP 96010-900 Capão-do-Leão, RS Brazil; 2 Empresa Brasileira de Pesquisa Agropecuária, Embrapa Uva e Vinho, CEP 95701-008 Bento Gonçalves, RS Brazil; 3 Empresa Brasileira de Pesquisa Agropecuária, Embrapa Clima Temperado, Caixa postal 403, CEP 96010-971 Pelotas, RS Brazil

**Keywords:** Developmental biology, Developmental biology, Ecology, Ecology

## Abstract

*Diachasmimorpha longicaudata* (Ashmead, 1905) (Hymenoptera: Braconidae) is considered one of the main biological control agents of *Ceratitis capitata* (Wiedemann) (Diptera: Tephritidae). However, the application of toxic baits for the management of *C*. *capitata* might exert side effects on the parasitoid. The objective of this study was to evaluate the side effects of toxic bait formulations on *D*. *longicaudata*. The food attractants Anamed, 3% Biofruit, 1.5% CeraTrap, 1.25% Flyral, 3% Isca Samaritá, 3% Isca Samaritá Tradicional, and 7% sugarcane molasses mixed with an organophosphate insecticide [malathion, 2.0 grams of active ingredient (g a.i.) L^−1^] and the commercial formulation Gelsura (2.0 and 4.0 g a.i. L^−1^ alpha-cypermethrin) showed high toxicity to *D*. *longicaudata* adults (>90% mortality) after 96 h and were thus classified as harmful (Class 4). Similarly, 3% Isca Samaritá Tradicional and 7% sugarcane molasses in formulations with the insecticides spinosad and spinetoram (0.096 g a.i. L^−1^ or kg) were moderately harmful (Class 3). In contrast, the food attractants Anamed, 3% Biofruit, 1.5% CeraTrap, 1.25% Flyral, and 3% Isca Samaritá Tradicional in combination with spinosad and spinetoram and the formulation Success 0.02CB (0.096 g a.i. L^−1^ spinosad) were classified as harmless (<10% mortality up to 96 h, Class 1). Additionally, these formulations did not reduce the parasitism and emergence rate of the F_1_ generation of *D*. *longicaudata* in *C*. *capitata* larvae. Formulations of toxic baits based on spinosyn are suitable for the management of *C*. *capitata* together with the parasitoid *D*. *longicaudata*.

## Introduction

The Mediterranean fruit fly, *Ceratitis capitata* (Wiedemann, 1824) (Diptera: Tephritidae), is a polyphagous and cosmopolitan species with a high capacity for infesting and damaging 361 host species belonging to 63 botanical families worldwide^1^. *C*. *capitata* has significant fruit-damaging potential because females of this species lay their eggs in fruit and the larvae subsequently open galleries in these fruits. The use of toxic baits has become one of the main strategies for the management of species populations worldwide^2–5^. The ingestion of toxic baits containing a lethal agent (insecticide molecule) mixed with a food attractant by adult Tephritidae causes mortality^6^.

Due to their broad spectrum and rapid action on fruit flies, organophosphate insecticides were once the most common insecticides in formulations of toxic baits^2,3^. However, because the use of broad-spectrum insecticides such as organophosphates needs to be avoided^3,6^, products derived from the fermentation of the bacterium *Saccharopolyspora spinosa* (Mertz and Yao) for the development of spinosyn insecticides (spinosad and spinetoram)^7^ are viable alternatives to organophosphates in toxic bait formulations^2,6–12^.

In Brazil, spinosyn-based insecticides are available in a concentrated suspension (Tracer 480 SC, spinosad), as a wettable powder (Spindle, spinosad), and as water-dispersible granules (Delegate 250 WG, spinetoram). Both insecticides are used in the formulation of toxic baits as an admixture with a protein and sugar-based food attractant^5^. A ready-to-use formulation (Success 0.02CB) was recently introduced in the Brazilian market. This formulation contains spinosad as the lethal agent, is internationally known as GF-120 NF^13^ and is registered for the management of *C*. *capitata* in mango crop.

Despite its low toxicity to mammals and fish, the insecticide spinosad can exert deleterious effects on the parasitism and longevity of different species of parasitoids after its ingestion^3,14,15^ and also exerts effects on the demographic traits of populations of natural enemies of parasitoids^16^.

The larval-pupal endoparasitoid *Diachasmimorpha longicaudata* (Ashmead, 1905) (Hymenoptera: Braconidae) has excellent potential for use in management programs for *C*. *capitata* in Brazil^17^. Factors linked to high parasitism and host-seeking capacity characterize the species as promising for large-scale multiplication and inundative releases in the field, and these strategies have been used in the United States, Guatemala, and Mexico^18,19^. Another important aspect is the biology of *D*. *longicaudata*, which is influenced by the size and age of the host, because larger *C*. *capitata* larvae in duce the production of higher amounts of *D*. *longicaudata* females. The effects of toxic bait formulations used in fruit-growing areas in Brazil on populations of *D*. *longicaudata* are unknown. Thus, this study aimed to evaluate the side effects of toxic bait formulations on *D*. *longicaudata* to ensure that these would not compromise integrated management programs for fruit flies.

## Results

### Toxicity on *D. longicaudata*

The food attractants Anamed, 3% Biofruit, 1.5% CeraTrap, 1.25% Flyral, and 7% sugarcane molasses showed low toxicity (<20% mortality) to *D*. *longicaudata* adults; the effects of these attractants were statistically similar (F_6, 72_ = 27.55, *P* < 0.0001) to those of the control (negative control, 15% mortality) after 96 h, and these attractants were thus classified as harmless (Tables 1 and 2). However, the food attractants 3% Isca Samaritá and 3% Isca Samaritá Tradicional resulted in more than 25% *D*. *longicaudata* mortality at 96 h, which was significantly different (*P* < 0.05) from the effects of the other food attractants, and these attractants were thus classified as slightly harmful to *D*. *longicaudata* adults (Tables 1 and 2). In toxic bait formulations, all food attractants mixed with the insecticide Malathion 1000 EC [2.0 gram of the active ingredient (g a.i.). L^−1^] showed higher toxicity (*P* < 0.05) to *D*. *longicaudata* adults during the first 24 h (mortality >85%) (48%) compared with the respective food attractant, and because 100% mortality was observed at 48 h, these formulations were classified as harmful (Class 4, Table 1). In contrast, the spinosyn-based insecticides (spinetoram and spinosad at a concentration of 0.096 g a.i. L^−1^ or kg) in an admixture with the food attractants Anamed, 3% Biofruit, 1.5% CeraTrap, 1.25% Flyral, and 3% Isca Samaritá Tradicional showed low toxicity at 96 h (<20% mortality), and their effects were statistically similar *(P* < 0.05) to those of the respective food attractants; thus, these insecticides were classified as harmless (Class 1, Table 3). However, 3% Isca Samaritá and 7% sugarcane molasses in an admixture with spinosad or spinetoram were classified as moderately harmful (Class 3) and harmful (Class 4), respectively, because their application resulted in more than 50% mortality at 24 h (Table 3). The ready-to-use toxic bait Success 0.02CB (0.96 g a.i. L^−1^ spinosad) caused low toxicity (up to 16.4% mortality at 96 h) to *D*. *longicaudata* adults and was classified as harmless (Class 1), and its effects were similar (*P* < 0.05) to those observed in insects fed a solution of water and 80% honey (Table 4). However, the exposure of *D*. *longicaudata* adults to the Gelsura formulation (2.0 and 4.0 g a.i. L^−1^ alpha-cypermethrin) resulted in higher than 90% mortality at 96 h, and this formulation was thus classified as harmful (Class 4).

**Table 1:** Food attractants in Brazil for the formulation of toxic baits used for fruit fly control.

Attractant	Description	Discriminatory concentration tested (% of dilution)^a^	Origin/Manufacturer
Anamed	40% SPLAT + 24.2% food attractant containing fruit extracts and phagostimulants	Without dilution	Isca Tecnologias Ltda., Ijuí, RS, Brazil
Biofruit	Hydrolyzed corn protein	3	BioControle Métodos de Controle de Pragas Ltda., Indaiatuba, São Paulo, Brazil
CeraTrap	Enzymatic hydrolyzed protein of animal origin	1.5	BioIbérica S.A., Barcelona, Spain
Flyral	Enzymatic hydrolyzed protein of animal origin	1.25	
Isca Samaritá	Hydrolyzed corn protein	3	Samaritá Indústria e Comércio Ltda., Artur Nogueira, São Paulo, Brazil
Isca Samaritá Tradicional	Vegetable protein, with reduced sugars and preservatives	3	
Surgarcane molasses	Byproduct with reduced sugars and non-crystallized sucrose	7	Originating from the sugar production process in the sugarcane industry

^a^Concentration (mL) of food attractant used in the formulation of the toxic baits.

**Table 2:** Mean number of alive insects (N ± Standard Error) and corrected mortality (%) of *D*. *longicaudata* when treated with toxic baits containing organophosphate insecticide and different food attractants.

Treatments	24 h		48 h		72 h		96 h		IOBC/WPRS Classe^c^
	N ± SE¹	M%²	N ± SE	M%	N ± SE	M%	N ± SE	M%	
Anamed + malathion 2.0 g i.a. L^−1^	0.9 ± 0.1 Aa	95.0	0.0 ± 0.0 Ab	100.0	0.0 ± 0.0 Ab	100.0	0.0 ± 0.0 Ab	100.0	4
Anamed	18.0 ± 0.4 Ba	0.0	17.5 ± 0.5 Ba	0.0	16.9 ± 0.5 Ba	0.0	16.5 ± 0.6 Ba	0.0	1
3% Biofruit + malathion 2.0 g i.a. L^−1^	1.9 ± 0.3 Aa	89.7	0.0 ± 0.0 Ab	100.0	0.0 ± 0.0 Ab	100.0	0.0 ± 0.0 Ab	100.0	4
3% Biofruit	18.4 ± 0.4 Ba	0.0	18.1 ± 0.0 Ba	0.0	18.1 ± 0.5 Ba	0.0	17.9 ± 0.4 Ba	0.0	1
1.5% CeraTrap + malathion 2.0 g i.a. L^−1^	0.7 ± 0.2 Aa	96.5	0.0 ± 0.0 Ab	100.0	0.0 ± 0.0 Ab	100.0	0.0 ± 0,0 Ab	100.0	4
1.5% CeraTrap 1.5%	20.0 ± 0.0 Ba	0.0	18.4 ± 0.0 Bab	0.0	16.7 ± 0.1 Bb	0.0	16.0 ± 0.2 Bb	0.0	1
1.25% Flyral + malathion 2.0 g i.a. L^−1^	0.9 ± 0.2 Aa	94.6	0.0 ± 0.0 Aa	100.0	0.0 ± 0.0 Aa	100.0	0.0 ± 0.0 Aa	100.0	4
1.25% Flyral 1.25%	16.8 ± 0.1 Ba	5.1	16.8 ± 0.1 Ba	0.0	16.4 ± 0.1 Ba	0.0	17.0 ± 0.1 Ba	0.0	1
3% Isca Samaritá + malathion 2.0 g i.a. L^−1^	0.7 ± 0.2 Aa	95.5	0.0 ± 0.0 Aa	100.0	0.0 ± 0.0 Aa	100.0	0.0 ± 0.0 Aa	100.0	4
3% Isca Samaritá	15.6 ± 0.2 Ba	11.9	11.3 ± 0.2 Bb	29.3	11.0 ± 0.2 Bc	31.5	10.6 ± 0.2 Bc	33.7	2
3% Isca Samaritá Tradicional + malathion 2.0 g i.a. L^−1^	1.2 ± 0.2 Aa	91.8	0.0 ± 0.0 Ab*	100.0	0.0 ± 0.0 Ab	100.0	0.0 ± 0.0 Ab	100.0	4
3% Isca Samaritá Tradicional	14.7 ± 0.1 Ba	16.9	13.6 ± 0.2 Ba	15.0	13.6 ± 0.2 Bab	15.0	11.4 ± 0.2 Bb	28.7	2
7% Sugarcane molasses + malathion 2.0 g i.a. L^−1^	1.2 ± 0.2 Aa	93.3	0.0 ± 0.0 Ab	100.0	0.0 ± 0.0 Ab	100.0	0.0 ± 0.0 Ab	100.0	4
7% Sugarcane molasses	17.8 ± 0.1 Ba	0.0	17.8 ± 0.1 Ba	0.0	17.4 ± 0.3 Ba	0.0	17.3 ± 0.3 Ba	0.0	1
80% Honey-water (negative control)	17.7 ± 0.5 Ba	—	16.0 ± 0.7 Ba	—	16.0 ± 0.7 Ba	—	16.0 ± 0.7 Ba	—	—

^a^Mean number of alive insects followed by the same uppercase letters in the same column do not differ significantly from each other when compared to the toxic bait formulation with the respective food attractant, and lowercase letters in the same row do not differ significantly from each other over time by Tukey’s test (*P* < 0.05).

^b^Mortality from the toxic bait corrected with the respective food attractant using the formula of Henderson and Tilton (1955)^33^.

^c^IOBC/WPRS class: Class 1 = harmless (M < 25%), Class 2 = slightly harmful (25% ≤ M ≤ 50%), Class 3 = moderately harmful (51% ≤ M ≤ 75%), and Class 4 = harmful (M > 75%).

**Table 3:** Mean number of alive insects (N ± Standard Error) and corrected mortality (%) of *D*. *longicaudata* when treated with toxic baits containing spinosyn-based insecticide and different food attractants.

Treatments	24 h		48 h		72 h		96 h		IOBC/WPRS Classe^c^
	N ± SE¹	M%²	N ± SE	M%	N ± SE	M%	N ± SE	M%	
Anamed + spinosad 0.096 g a.i. L^−1^	17.2 ± 0.5 Ba	6.0	15.1 ± 0.8 Bab	11.7	15.0 ± 0.7 Bab	11.2	14.2 ± 0.8 Bb	12.9	1
Anamed + spinetoram 0.096 g a.i. kg	16.9 ± 0.7 Ba	7.6	14.7 ± 0.9 Bb	14.0	14.4 ± 0.9 Bb	14.8	14.0 ± 0.8 Bb	14.1	1
Anamed	18.3 ± 0.4 Ba	0.0	17.1 ± 0.5 Ba	0.0	16.9 ± 0.5 Ba	0.0	16.3 ± 0.6 Ba	0.0	1
3% Biofruit + spinosad 0.096 g a.i. L^−1^	17.8 ± 0.5 Ba	0.0	16.8 ± 0.6 Ba	0.0	16.3 ± 0.7 Ba	0.0	15.3 ± 0.7 Ba	0.0	1
3% Biofruit + spinetoram 0.096 g a.i. kg	19.4 ± 0.2 Ba	0.0	18.5 ± 0.3 Ba	0.0	18.5 ± 0.3 Ba	0.0	18.5 ± 0.3 Ba	0.0	1
3% Biofruit	17.4 ± 0.6 Ba	1.7	15.6 ± 0.7 Bab	0.0	15.3 ± 0.8 Bab	1.9	15.1 ± 0.7 Bb	0.0	1
1.5% CeraTrap + spinosad 0.096 g a.i. L^−1^	15.8 ± 0.6 Ba	14.1	14.6 ± 0.5 Bab	9.3	14.3 ± 0.6 Bab	5.9	12.9 ± 0.8 Bb	11.6	1
1.5% CeraTrap + spinetoram 0.096 g a.i. kg	18.8 ± 0.3 Ba	0.0	18.2 ± 0.4 Ba	0.0	18.0 ± 0.3 Ba	0.0	17.8 ± 0.6 Ba	0.0	1
1.5% CeraTrap	18.4 ± 0.6 Ba	0.0	16.1 ± 0.7 Bab	0.0	15.2 ± 0.7 Bb	2.6	14.6 ± 0.8 Bb	3.3	1
1.25% Flyral + spinosad 0.096 g a.i. L^−1^	14.8 ± 0.9 Ba	17.3	13.7 ± 1.0 Bab	13.8	12.9 ± 1.1 Bb	15.7	12.0 ± 1.2 Bb	19.5	1
1.25% Flyral + spinetoram 0.096 g a.i. kg	15.4 ± 1.0 Ba	13.9	14.1 ± 1.0 Bab	11.3	13.6 ± 0.9 Bab	11.1	13.0 ± 0.9 Bb	12.7	1
1.25% Flyral	17.9 ± 0.5 Ba	0.0	15.9 ± 1.4 Ba	0.0	15.3 ± 1.4 Ba	1.9	14.9 ± 1.5 Ba	1.3	1
3% Isca Samaritá + spinosad 0.096 g a.i. L^−1^	6.4 ± 0.6 Aa	58.2	5.7 ± 0.5 Aa	52.5	5.1 ± 0.5 Aa	54.0	5.0 ± 0.5 Aa	51.9	3
3% Isca Samaritá + spinetoram 0.096 g a.i. kg	4.3 ± 0.5 Aa	71.9	3.6 ± 0.6 Aa	70.0	3.0 ± 06 Aa	73.0	2.8 ± 0.6 Aa	73.1	3
3% Isca Samaritá	15.3 ± 0.7 Ba	13.6	12.0 ± 0.5 Bab	23.1	11.1 ± 0.4 Bb	28.8	10.4 ± 0.5 Bb	31.3	2
3% Isca Samaritá Tradicional + spinosad 0.096 g a.i. L^−1^	15.2 ± 0.7 Ba	2.5	13.1 ± 0.7 Ba	3.7	13.1 ± 0.7 Ba	3.7	11.7 ± 0.7 Ba	0.0	1
3% Isca Samaritá Tradicional + spinetoram 0.096 g a.i. kg	15.6 ± 0.6 Ba	0.0	14.8 ± 0.6 Bab	0.0	14.1 ± 0.5 Bab	0.0	13.1 ± 0.4 Bb	0.0	1
3% Isca Samaritá Tradicional	14.7 ± 0.1 Ba	16.9	13.6 ± 0.2 Ba	15.0	13.6 ± 0.2 Ba	15.0	11.4 ± 0.2 Bb	28.7	2
7% Sugarcane molasses + spinosad 0.096 g a.i. L^−1^	4.8 ± 0.4 Aa	74.7	4.5 ± 0.4 Aa	73.2	4.2 ± 0.5 Aa	73.9	4.1 ± 0.5 Aa	74.3	3
7% Sugarcane + spinetoram 0.096 g a.i. kg	2.6 ± 0.4 Aa	86.2	2.5 ± 0.3 Aa	85.1	2.3 ± 0.3 Aa	85.7	2.3 ± 0.3 Aa	85.7	4
7% Sugarcane	18.8 ± 0.3 Ba	0.0	16.8 ± 0.5 Ba	0.0	16.1 ± 0.7 Ba	0.0	16.1 ± 0.7 Ba	0.0	1
80% Honey-water solution (negative control)	17.7 ± 0.5 Ba	—	15.6 ± 0.7 Ba	—	15.6 ± 0.7 Ba	—	15.1 ± 0.7 Ba	—	—

^a^Mean number of alive insects followed by the same uppercase letters in the same column do not differ significantly from each other when compared to the toxic bait formulation with the respective food attractant, and lowercase letters in the same row do not differ significantly from each other over time by Tukey’s test (*P* < 0.05).

^b^Mortality from the toxic bait corrected with the respective food attractant using the formula of Henderson and Tilton (1955)^33^.

^c^IOBC/WPRS class: Class 1 = harmless (M < 25%), Class 2 = slightly harmful (25% ≤ M ≤ 50%), Class 3 = moderately harmful (51% ≤ M ≤ 75%), and Class 4 = harmful (M > 75%).

**Table 4:** Mean number of live insects (N ± Standard Error) and corrected mortality (%) of *D*. *longicaudata* when submitted to treatment with ready-to-use toxic baits.

Treatments	24 h		48 h		72 h		96 h		IOBC/WPRS Classe^c^
	N ± SE^a^	M%²	N ± SE	M%	N ± SE	M%	N ± SE	M%	
Success 0.02CB (spinosad 0.096 g a.i. L^−1^)	16.4 ± 0.7 Ba	7.3	13.4 ± 0.8 Bb	14.1	13.1 ± 1.0 Bb	16.0	12.6 ± 1.0 Bb	16.5	1
Gelsura (alpha-cypermethrin 2.0 g a.i. L^−1^)	1.1 ± 0.4 Aa	93.8	0.9 ± 0.4 Aa	94.2	0.9 ± 0.4 Aa	94.2	0.7 ± 0.3 Aa	95.4	4
Gelsura (alpha-cypermethrin 4.0 g a.i. L^−1^)	3.1 ± 1.8 Aa	82.5	2.2 ± 0.8 Aab	85.9	1.6 ± 0.5 Ab	89.7	1.4 ± 0.2 Ab	90.7	4
80% Honey-water solution (negative control)	17.7 ± 0.52 Ba	—	15.6 ± 0.7 Bab	—	15.6 0.7 Bab	—	15.1 ± 0.7 Bb	—	—

^a^Mean number of alive insects followed by uppercase letters in the same column and lowercase letters in the same row do not differ significantly from each other by Tukey’s test (*P* > 0.05).

^b^Mortality corrected with the negative control using the formula of Henderson and Tilton (1955)^33^.

^C^IOBC/WPRS class: 1) harmless (M < 30%), 2) slightly harmful (30 ≤ M ≤ 79%), 3) moderately harmful (80 ≤ M ≤ 99%), and 4) harmful (M > 99%).

### Side effects on *D*. *longicaudata*

Because the food attractants Anamed, 3% Biofruit, 1.5% CeraTrap, 1.25% Flyral, and 3% Isca Samaritá Tradicional alone or in an admixture with the insecticide spinosad or spinetoram, 3% Isca Samaritá in an admixture with spinosad and the Success 0.02CB formulation showed low toxicity (<50% mortality) on *D*. *longicaudata* adults, the coefficients for the reductions in the parasitism or emergence of the parasitoid on *C*. *capitata* larvae were estimated. The results showed that the exposure of *D*. *longicaudata* adults to Anamed, 3% Biofruit, 1.5% CeraTrap, and 1.25 Flyral alone or in admixture with the insecticide spinosad or spinetoram (0.096 g a.i. L^−1^ or kg) for 24 h did not significantly (*P* < 0.05) reduce the parasitism and adult emergence rates of *C*. *capitata* larvae (<30% reductions) compared with the negative control (80% honey-water solution). Therefore, these insecticides were classified as harmless (Class 1) with respect to both evaluated responses (Table 5). In contrast, 3% Isca Samaritá Tradicional alone or in an admixture with spinosad or spinetoram exerted significant side effects in *D*. *longicaudata* adults. Specifically, both treatments induced reductions in parasitism (F_16, 119_ = 9.11, *P* < 0.0001) and insect emergence (F_16, 125_ = 4.06, *P* < 0.0001) compared with the negative control (Table 5), and these formulations were thus classified as slightly harmful (Class 2).

**Table 5:** Side effects of food attractants and toxic bait formulations in *D*. *longicaudata* adults.

Treatments	% Parasitism^*a*^	RP	IOBC/WPRS class	Emerged insects^*a*^	RE	IOBC/WPRS class
Anamed + spinosad 0.096 g a.i. L^−1^	80.8 ± 10.6*	0.0	1	91.0 ± 11.3*	26.7	1
Anamed + spinetoram 0.096 a.i. kg	83.5 ± 6.6*	0.0	1	122.7 ± 25.6	1.2	1
Anamed	53.7 ± 2.2	11.6	1	123.0 ± 11.2	0.9	1
3% Biofruit + spinosad 0.096 g a.i. L^−1^	79.2 ± 12.8*	0.0	1	132.6 ± 18.1	0.0	1
3% Biofruit + spinetoram 0.096 g a.i. kg	64.1 ± 7.3	0.0	1	113.0 ± 5.6	8.9	1
3% Biofruit	75.2 ± 15.1*	0.0	1	102.0 ± 14.9*	17.8	1
1.5% CeraTrap + spinosad 0.096 g a.i. L^−1^	67.5 ± 7.5	0.0	1	87.3 ± 6.0*	29.8	1
1.5% CeraTrap + spinetoram 0.096 g a.i. kg	78.3 ± 8.7*	0.0	1	123.6 ± 7.4	0.4	1
1.5% CeraTrap	77.6 ± 7.5*	0.0	1	123.2 ± 7.1	0.7	1
1.25% Flyral + spinosad 0.096 g a.i. L^−1^	83.03 ± 3.4*	0.0	1	134.2 ± 20.4	2.9	1
1.25% Flyral + spinetoram 0.096 g a.i. kg	56.0 ± 7.8	7.6	1	120.6 ± 12.7	0.4	1
1.25% Flyral	63.7 ± 5.3	0.0	1	111.7 ± 14.0	10.0	1
Isca SamaritáTradicional 3% + spinosad 0.096 g a.i. L^−1^	32.7 ± 4.4*	32.1	2	64.1 ± 32.0*	48.4	2
Isca Samaritá Tradicional 3% + spinetoram 0.096 g a.i. kg	35.6 ± 5.4*	41.3	2	50.2 ± 14.6*	59.6	2
Isca Samarita Tradicional 3%	22.9 ± 1.1*	62.2	2	72.8 ± 7.7*	58.6	2
Success 0,02CB (spinosad 0.096 g i.a. L^−1^)	72.9 ± 7.2*	0.0	1	138.2 ± 6.7	0.0	1
80% Honey-water solution (negative control)	60.7 ± 2.6	—	—	124.2 ± 10.1	—	—

Mean number of alive insects ± standard error (N ± SE).

^*^Significantly different relative to the negative control and from each other according to Tukey’s test (*P* < 0.05).

RP = Reduction of parasitism and RE = Reduction of emergence.

IOBC/WPRS class: harmless (RP or RE < 30%); 2) slightly harmful (30 ≤ RP or RE ≤ 79%); 3) moderately harmful (80 ≤ RP or RE ≤ 99%), and 4) harmful (RP or RE > 99%).

## Discussion

Based on the isolated effects of the food attractants, 3% Isca Samaritá and 3% Isca Samaritá Tradicional were classified as moderately toxic to *D*. *longicaudata* adults. Additionally, these attractants negatively affected the rates of parasitism and emergence of the insects in F_1_ *C*. *capitata* larvae compared with a solution of water and 80% honey, which is considered the standard for laboratory rearing and multiplication of the species^17^. The same finding was not obtained with Anamed, 3% Biofruit, 1.5% CeraTrap, 1.25% Flyral, and 7% sugarcane molasses, which might be associated with the higher protein and carbohydrate composition of these attractants compared with that of 3% Isca Samaritá and 3% Isca Samaritá Tradicional. In this study, the amount of food attractant ingested by the insects during the exposure time (24 h) was not measured. However, *D*. *longicaudata* adults were attracted to and fed on all treatments offered.

High *D*. *longicaudata* mortality rates were measured after a short period (24 h) of feeding with the attractants 3% Isca Samaritá and 3% Isca Samaritá Tradicional and the mixtures without insecticide. This finding might indicate that these attractants do not provide the nutrients (carbohydrates) necessary for the survival of the individuals during the first days of life. According to previous studies, this period is considered crucial for the development and maturation of the ovaries, which is necessary for reproduction of the species^20,21^. In addition, although its detailed composition is not available from the manufacturer, the vegetable protein obtained from sugarcane can likely undergo a fermentation process after the addition of water, resulting in the generation of byproducts that are toxic to *D*. *longicaudata*, such as ethanol. Similar findings have been reported for *Drosophila melanogaster* Meigen, 1830 (Diptera: Drosophilidae) against Figitidae parasitoid wasps^22,23^.

The highest toxicity against *D*. *longicaudata* adults was obtained with the toxic baits formulated with the insecticide Malathion 1000 EC (2.0 g a.i. L^−1^) in an admixture with Anamed, 3% Biofruit, 1.5% CeraTrap, 1.25% Flyral, 3% Isca Samaritá, 3% Isca Samaritá Tradicional, and 7% sugarcane molasses (Class 4). *D*. *longicaudata* adults fed toxic baits containing an organophosphate insecticide showed high nervous hyperactivity “tremors” within a few hours of feeding. Due to its high toxicity to fruit fly species, organophosphate insecticides have commonly been used in the formulation of toxic baits^3,11,24^. Additionally, the ease of acquisition and the low cost of the active ingredients constitute advantages to fruit growers in rural properties that utilize toxic bait formulations^5^.

Toxic baits formulated with the organophosphate insecticide malathion were compared with the ready-to-use formulation Gelsura (2.0 and 4.0 g a.i. L^−1^ alpha-cypermethrin), which is under evaluation in Brazil for the management of *Anastrepha fraterculus* (Wiedemann, 1830) (Diptera: Tephritidae) and *C*. *capitata*^5^. Studies carried out in the Mediterranean region have demonstrated the potential of use of this formulation for the population suppression of *Bactrocera oleae* (Rossi, 1790) (Diptera: Tephritidae) in olive crops and *C*. *capitata* in citrus^25^. To date, scarce information on the toxicity and side effects of this formulation on natural enemies has been reported. The present study demonstrated that the toxic bait resulted in high mortality of *D*. *longicaudata* adults during the first 24 h after ingestion, leading to its classification as harmful (Class 4). This result might be associated with the knockdown effect caused by the presence of the pyrethroid insecticide alpha-cypermethrin at 0.2%, as observed in preliminary tests with *A*. *fraterculus* and *C*. *capitata* (unpublished data).

In contrast, toxic baits formulated with a food attractant plus spinosad (Tracer 480 SC) or spinetoram (Delegate 250 WG) at a concentration of 0.096 g a.i. L^−1^ or kg, which is equivalent to the concentration in the ready-to-use formulation Success 0.02CB, showed low toxicity (<10% mortality) on *D*. *longicaudata*. Studies conducted in other regions also showed that toxic baits containing the insecticide spinosad exert reduce effects on biological control agents. An evaluation of the effect of the GF-120 NF bait on *D*. *longicaudata* through cage bioassays with mango plants resulted in less than 40% mortality^3^.

Similarly, toxic bait formulations containing spinosad are less toxic to the parasitoids *Fopius arisanus* (Sonan, 1932) and *Psyttalia fletcheri* (Silvestri, 1916) (Hymenoptera: Braconidae) than toxic baits formulated with the insecticide malathion^3^. As verified in Hawaii, the field application of 11 sprayings of toxic baits containing the insecticide spinosad (GF-120 NF) had no effects on the *F*. *arisanus* population. However, the toxicity of spinosyn-based insecticides depends on the manner of application (ingestion or topical)^15^, the dose and time of exposure and the insect species being studied^3,26^.

In addition, toxic baits containing spinosyn-based insecticides (spinosad and spinetoram) and the ready-to-use formulation Success 0.02CB exerted no side effects on parasitism. Additionally, the mean number of offspring in the F_1_ generation after 7 consecutive days of parasitism in *C*. *capitata* larvae fed these formulations was similar to that obtained with the 80% honey solution^17^. The feeding of *D*. *longicaudata* adults with the toxic bait GF-120 NF in an admixture with honey for 24 h exerted no side effects on the progeny. However, the daily feeding of the toxic bait GF-120 NF and honey resulted in no significant reductions in the offspring number^3^.

The Integrated Control of Noxious Animals and Plants (IOBC) has promoted studies aiming to standardize selectivity tests and thereby reduce problems related to differences in methodology. Nevertheless, diverging results continue to be reported for many reasons^27^. The classification of a specific product (used at the same rate) can range from harmless to harmful depending on the developmental stage of the natural enemy, and differences (e.g., in body size and sex ratio; different tolerances have been found between males and females) can be detected between a specific species and the natural enemies of that species^27^. Thus, a better understanding of the diversity of biological control species in agroecosystems and of the different mechanisms through which pesticides can affect the effectiveness of these species is needed.

The new challenge in the study of the selectivity of pesticides for natural biocontrol agents is to go beyond the description of lethal/sublethal effects of pesticides on natural control agents. Similarly, the ecological structure within agroecosystems should be considered. The results obtained in this study are based on criteria directly obtained from the IOBC with respect to the side effects of insecticides on natural enemies. The above-described context can be very simple and might differ from what would occur in field conditions. As a result, the criteria provided by the IOBC should be improved to aid the classification of insecticides and toxic bait formulations with respect to their natural enemies^27^.

The results for the different toxic baits included in this study were obtained in the laboratory, where the *D*. *longicaudata* adults were caged and forced to feed on the material offered. Thus, the side effects on *D*. *longicaudata* in the field might be less damaging. This possibility is associated with the low biological persistence of toxic baits when applied in the field because the active ingredient is degraded by the presence of light or constant rainfall^3,10,11^. In addition, parasitoids can avoid contact with toxic baits and look for other food sources, as has been observed with *F*. *arisanus*^28,29^. Based on this finding, it is also important to evaluate other bioassay methodologies for assessing the effects on parasitoids, as proposed in a previous study^30^. Given these results, fruit flies can be managed by combining chemical control through the application of spinosyn-based toxic baits with biological control using *D*. *longicaudata* for the suppression of pest populations. However, new studies should be performed under semi-field and field conditions to obtain a better understanding of the effect of the tested formulations on the parasitoids.

## Materials and Methods

### Insect rearing

*C*. *capitata* and *D*. *longicaudata* adults were obtained from the Entomology Laboratory of Embrapa Temperate Climate, Pelotas, RS, Brazil, and were maintained in air-conditioned rooms (temperature of 25 ± 2 °C, 70 ± 10% relative air humidity, and 12-h photoperiod). *C*. *capitata* adults were obtained from mango fruits (*Mangifera indica* L.) collected in the municipality of Pelotas, Rio Grande do Sul, Brazil (31°38′20″ S and 52°30′43″ W), infested with *C*. *capitata* larvae and were established and maintained (20 generations) in the laboratory on an artificial diet prior to their use in the bioassay^31^. In the laboratory, the adults were kept in plastic cages (57-cm long × 39-cm wide × 37-cm high; Sanremo, Bettanin Industrial S/A) and were provided distilled water supplied via capillarity from hydrophilic cotton and an artificial diet based on refined sugar, wheat germ, and beer yeast (Bionis YE NS + MF) (3: 1: 1 ratio), which was supplied in a Gerbox germination box (11-cm long × 11-cm wide × 3.5-cm high)^31^. The methodology proposed in a previous study^29^ was used for the preparation of the rearing and egg collection cages. The collected eggs were transferred to Erlenmeyer glass containers (500 mL) and aerated for 24 h. After this period, the eggs (≈9,200) were deposited on a strip of filter paper (10 cm^2^) using a micropipette (30 μL) and placed in plastic containers (17-cm wide × 27.6-cm long × 7-cm high) over a layer of artificial diet (300 mL) for larval development^32^. After nine days, the artificial diet was removed, and the larvae were collected using a sieve (0.22-mm mesh) and packed in plastic containers (300 mL) on a layer of moistened fine vermiculite (1 cm), where pupation and emergence of the adults occurred.

*D*. *longicaudata* individuals were obtained from field collections of *C*. *capitata* larvae in the municipality of Pelotas, RS, Brazil. In the laboratory, the insects were maintained for approximately 30 generations prior to their use in the bioassay and maintained in plastic cages (45-cm long × 30-cm wide × 30-cm high)^17^. The adults were fed an 80% honey aqueous solution supplied via capillarity using a strip of sponge (Spontex) placed inside a Petri dish (3 cm in diameter). Seven days after emergence, third instar larvae from reared *C*. *capitata* were exposed to parasitism for 1 h (30 larvae per female *D*. *longicaudata*). After this period, the larvae were removed and maintained on a layer of fine vermiculite (1 cm) in plastic containers (200 mL), and the top of each container was closed with a lid to enable pupation and the emergence of adult parasitoids. The *D*. *longicaudata* adults used in the bioassays were collected from *C*. *capitata* maintained under laboratory conditions for approximately 20 generations.

### Bioassays and toxic bait formulations

To evaluate the toxicity and side effects of the toxic bait formulations on *longicaudata* adults, ingestion bioassays were performed in an air-conditioned room (temperature of 25 ± 2 °C, 70 ± 10% relative humidity, and 12-h photoperiod). The tested food attractants were Anamed, 3% Biofruit, 1.5% CeraTrap, 1.25% Flyral, 3% Isca Samaritá, 3% Isca Samaritá Tradicional and 7% sugarcane molasses (Table 1). The concentrations of these food attractants were defined based on the manufacturer’s recommendations and practical experience. The following insecticides were used to formulate toxic baits based on admixtures containing the above-mentioned food attractants: Malathion 1000 EC (1.0 g a.i. L^−1^ malathion; Cheminova Ltda., São Paulo, Brazil), Tracer 480 SC (480 g a.i. L^−1^ spinosad) and Delegate 250 WG (250 g a.i. kg spinetoram; Dow AgroSciences Industrial Ltda., São Paulo, Brazil). These products are registered for the management of fruit flies in Brazil.

The ready-to-use formulations used in this study were Success 0.02CB [0.24 g a.i. L^−1^ spinosad; Dow AgroSciences Industrial Ltda., São Paulo, Brazil; diluted in water at a ratio of 1:1.5 volume/volume (v/v), i.e., 1 part of the commercial product to 1.5 parts of water] and Gelsura [6.0 g a.i. L^−1^ alpha-cypermethrin, a polymer matrix containing the active ingredient alpha-cypermethrin; BASF SA, São Paulo, Brazil; diluted 1:2 and 2:1 (parts of the commercial product:parts of water)]. A solution containing 80% honey and water was used as the negative control for both treatments.

A completely randomized experimental design with 32 treatments and 10 replicates per treatment (food attractant + insecticide, only food attractant, or 80% honey and water solution) and 10 *D*. *longicaudata* (*n* = 100) couples per treatment was used in this study.

### Toxicity toward *D*. *longicaudata* adults

Three-day-old insects from the rearing cage were starved for 12 h. At the end of this period, 10 couples were placed inside a cage consisting of a plastic container (500 mL) inverted on an acrylic plate (12 cm in diameter). The top of the case was cut out and covered with a fine mesh net to allow ventilation. Subsequently, one drop (10 mm) of each treatment was placed with a micropipette (100 μL) on a plastic plate (2.5 cm in diameter) composed of plastic paraffin film and parafilm paper (Bemis Company, Inc., USA). The insects were allowed to feed on the treatments for a period of 24 h. Subsequently, the insects were fed an 80% honey aqueous solution supplied via capillarity as previously described. The numbers of insects that were still alive or had died after 24, 48, 72, and 96 h of exposure were recorded. The insects that showed no reaction to touching with a fine-tipped brush were considered dead. To isolate the effect of each food attractant on *D*. *longicaudata* adults, the mortality of each toxic bait formulation (food attractant + insecticide) was corrected to that obtained with the corresponding food attractant using the formula developed by Henderson and Tilton^33^. Similarly, the mortality caused by the food attractant was corrected with that obtained with the negative control (water and 80% honey solution). Based on the mortality (M) data at 96 h, the treatments were classified according to the criteria defined by IOBC/WPRS as follows: Class 1 = harmless (M < 25%), Class 2 = slightly harmful (25% ≤ M ≤ 50%), Class 3 = moderately harmful (51% ≤ M ≤ 75%), and Class 4 = harmful (M > 75%)^34^.

### Side effects on *D*. *longicaudata* adults

The treatments that caused less than 50% mortality in a population of *D*. *longicaudata* adults (Classes 1 and 2) were used. Adults (10 couples per cage, 3 days of age) were fasted for a period of 12 h and used for the assessment of adult toxicity as described. After 24 h of treatment, the surviving adults were fed a mixture of water and 80% honey until the end of the bioassay. Starting on the seventh day after emergence, third instar larvae of *C*. *capitata* (30 larvae per female) were offered the treatment daily for seven consecutive days^17^. After 1 h of daily parasitism, the larvae were removed and stored in plastic containers (100 mL) containing a layer of fine vermiculite (1 cm) until adult emergence. After emergence of the first insect (*C*. *capitata* or *D*. *longicaudata*), the puparia were evaluated daily. At the end of the bioassay, pupae that remained intact were dissected to assess the presence of nonemergent flies or parasitoids and tthus determine the true parasitism rate. The reductions in the parasitism capacity (%) and emerged insects (%) obtained with each treatment were determined in comparison with the negative control and calculated using the formula RP = [(1 − T/C) * 100], where T is the mean parasitism or mean emergence with the treatment (the toxic bait formulation or the food attractant alone) and C is the mean parasitism or emerged insects obtained with the negative control (water + honey solution)^35^. Based on the obtained reductions in the parasitism (% RP) and emergence (% RE) of *D*. *longicaudata*, the treatments were classified according to the IOBC as follows: harmless (RP or RE < 30%), slightly harmful (30 ≤ RP or RE ≤ 79%), moderately harmful (80 ≤ RP or RE ≤ 99%), and harmful (RP or RE > 99%).

### Statistical analysis

The data were initially subjected to residual analysis to confirm the assumption of normality obtained with the Shapiro-Wilk test and to an analysis of the variance homogeneity based on the Bartlett test using the PROC UNIVARIATE procedure in SAS 9.1^36^. The survival rates of the *D*. *longicaudata* adults that did not present a normal distribution were subjected to Box-Cox transformation prior to the analyses. Subsequently, all the data were subjected to a two-way analysis of variance using PROC GLM^36^. The differences between the treatments were determined by the least-squares means (PDIFF option in PROC GLM) followed by Tukey’s adjustment based on a 5% significance threshold using SAS 9.1 software^36^. To evaluate the side effects on *D*. *longicaudata* adults, the data on parasitism (%) and emergence (%) were evaluated for normality using the Shapiro-Wilk test and homoscedasticity using the Hartley and Bartlett test and then subjected to analysis of variance (ANOVA option in PROC GLM) using the F test (*P* < 0.05)^36^. When statistically significant, the means were compared by Tukey’s test at the 5% significance level (*P* < 0.05).

### Data archiving

This article does not report new empirical data or software.
